# Application of 3D Bioprinting Technologies to the Management and Treatment of Diabetic Foot Ulcers

**DOI:** 10.3390/biomedicines8100441

**Published:** 2020-10-21

**Authors:** Chew Teng Tan, Kun Liang, Zong Heng Ngo, Christabel Thembela Dube, Chin Yan Lim

**Affiliations:** 1 Skin Research Institute of Singapore (SRIS), Agency for Science, Technology and Research (A*STAR), Singapore 138648, Singapore; tan_chew_teng@sris.a-star.edu.sg (C.T.T.); kun_liang@sris.a-star.edu.sg (K.L.); zongheng00@gmail.com (Z.H.N.); christabel_thembela_dube_from.tp@sris.a-star.edu.sg (C.T.D.); 2 Faculty of Biology, Medicine and Health, University of Manchester, Manchester M13 9PT, UK; 3 Department of Biochemistry, Yong Loo Lin School of Medicine, National University of Singapore, Singapore 117596, Singapore

**Keywords:** 3D bioprinting, diabetic foot ulcers, wound healing

## Abstract

Diabetes mellitus (DM) is a chronic metabolic disease with increasing prevalence worldwide. Diabetic foot ulcers (DFUs) are a serious complication of DM. It is estimated that 15–25% of DM patients develop DFU at least once in their lifetime. The lack of effective wound dressings and targeted therapy for DFUs often results in prolonged hospitalization and amputations. As the incidence of DM is projected to rise, the demand for specialized DFU wound management will continue to increase. Hence, it is of great interest to improve and develop effective DFU-specific wound dressings and therapies. In the last decade, 3D bioprinting technology has made a great contribution to the healthcare sector, with the development of personalized prosthetics, implants, and bioengineered tissues. In this review, we discuss the challenges faced in DFU wound management and how 3D bioprinting technology can be applied to advance current treatment methods, such as biomanufacturing of composite 3D human skin substitutes for skin grafting and the development of DFU-appropriate wound dressings. Future co-development of 3D bioprinting technologies with novel treatment approaches to mitigate DFU-specific pathophysiological challenges will be key to limiting the healthcare burden associated with the increasing prevalence of DM.

## 1. Introduction

Diabetes mellitus (DM) is a chronic metabolic disease with increasing prevalence worldwide [1,2,3,4,5,6]. DM is characterized by persistent hyperglycemia associated with the lack of insulin production (Type 1 DM) or insulin resistance (Type 2 DM) [7,8]. In the long term, DM leads to a decline in the physiological function of organ systems such as the integumentary, nervous and immune systems, resulting in serious health complications [9,10,11,12]. Diabetic foot ulcers (DFUs) are one of the most common complications in diabetic patients. Approximately 15–25% of diabetic patients will develop a DFU that requires extensive wound management at least once in their lifetime [6,13]. Although some cases of DFUs are reported to heal successfully, a significant number of DFUs fail to improve with wound management. As a result, lower limb amputation is often performed to prevent sepsis and other complications resulting from infections at the wound site [4,5,13]. DFUs have a significant impact on the quality of life in diabetic patients due to pain, use of multidrug treatment regimens, prolonged hospitalization, decreased mobility, and increased mortality rate [3,14,15]. The number of people living with DM is projected to reach 592 million by 2030 and without a solution, the prevalence of DFUs is expected to rise [16]. This highlights a major public health concern and an economic burden due to the cost of implementing long-term healthcare systems for diabetic patients. Hence, it is imperative to improve current treatments and develop new therapeutic tools to treat DFUs.

Advanced 3D bioprinting technologies have, in recent years, accelerated development in healthcare areas such as personalized prosthetics, implants and tissue fabrication [17,18,19,20]. In this review, major differences between acute and chronic wound healing process are first highlighted with an emphasis on the pathophysiology of DFUs and the impact of high glucose microenvironment on wound healing that distinguishes DFUs from other chronic wounds. The application of 3D bioprinting technology to improve wound management of DFUs, particularly in enhancing skin grafting procedures with the use of personalized 3D human skin constructs as well as the development of DFU-specific wound dressing products, is then discussed.

## 2. The Human Skin Structure

The skin is a specialized organ that provides a barrier between the body and its external environment. The skin and its appendages, including hair follicles, eccrine and sebaceous glands, form the integumentary system, involved in immune surveillance, thermoregulation, protection from ultraviolet light and vitamin D synthesis [21,22,23]. The skin is made up of three main layers—the epidermis, the dermis and the hypodermal layer. The outermost layer of the skin, the epidermis, is made up of four distinct successive sublayers that act as a permeability barrier of the skin, preventing excessive water loss from the body, and protecting against chemical and physiological insults [21,24]. Located beneath the epidermis is the dermis which is characterized by the presence of collagen and elastic fibres, fibroblasts, extracellular matrix (ECM), blood vessels and nerves. The dermis is made up of two sublayers, the papillary and the reticular layer. The papillary layer consists of dermal papillae that interlink with the epidermal ridges, forming the dermal–epidermal junction, thereby increasing the strength and stability of the skin. The reticular layer contains an extensive supply of blood vessels that provide the skin with nutrients and oxygen [22,25]. Also resident within the reticular layer of the dermis are the skin’s appendages and numerous cells of the innate immune system, such as macrophages, mast cells and dermal dendritic cells [26]. The third main layer of the skin is the subcutaneous fat layer (hypodermis). It protects the body from physical trauma and also functions as an insulator for the tissues underlying the skin [25].

## 3. Normal and Chronic Wound Healing

**Figure 1:**
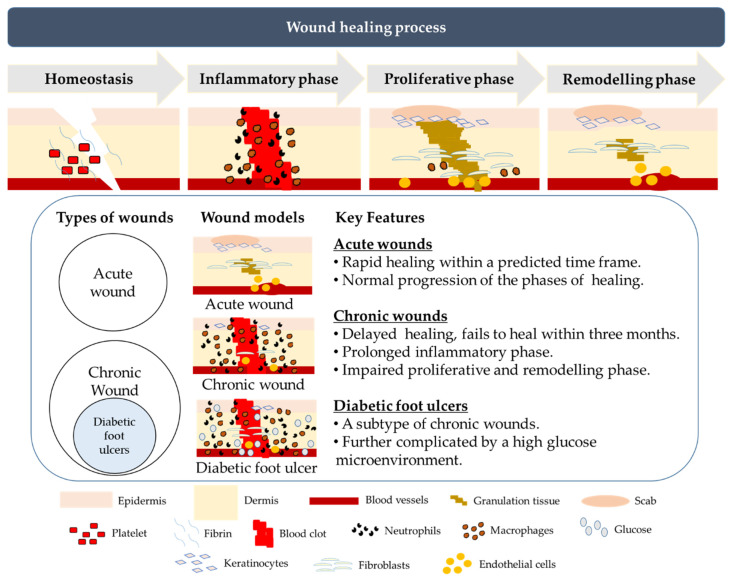
An illustration of the different phases of cutaneous wound healing and the key differences between acute wounds, chronic and diabetic foot ulcers. In normal wounds, healing is initiated by hemostasis to minimize further blood loss, followed by the inflammatory phase to prevent microbial infection. In the proliferative phase, granulation tissue is formed accompanied by the proliferation of epidermal keratinocytes and dermal fibroblasts, facilitating the final phase of tissue remodeling to restore the structure and function of the skin. Wounds can be classified as acute and chronic. Acute wounds heal rapidly within a short time frame with normal progression of the phases of healing, while chronic wounds take more than three months to heal due to a prolonged inflammatory phase and impaired proliferative and remodeling phases. Diabetic foot ulcers exhibit similar characteristics as other chronic wounds, but the healing of diabetic foot ulcers (DFUs) is further complicated by a sustained high glucose microenvironment.

Cutaneous wound healing refers to the complex physiological process of restoring the skin’s integrity and function following damage by internal and external insults such as infections and burns. Successful wound healing is achieved through the optimal progression of four temporally overlapping healing phases: hemostasis; inflammation; proliferation; and remodeling (Figure 1) [27]. The initial phase of healing, hemostasis, occurs immediately after an injury to prevent excessive bleeding and to maintain continuous blood flow within the rest of the circulatory pathway [28,29]. The hemostatic process of wound healing is accompanied by a protective inflammatory response targeted at preventing further infiltration of the wounded area by external microorganisms and sterilizing the wound in preparation for healing. Tissue-resident macrophages in the skin secrete pro-inflammatory chemokines, such as interleukin-8 (IL-8/ CXCL8) and monocyte chemoattractant protein-1 (MCP-1/CCL2), which coordinate the migration of neutrophils and monocytes towards the injured area [30]. Neutrophils that are normally circulating in the blood are extravasated through the endothelial wall to enter the interstitial fluid and migrate towards the wound bed [31,32]. Inflammatory signals then transform monocytes arriving at the site of injury into pro-inflammatory macrophages that phagocytose bacteria, dead neutrophils and damaged cells [33,34]. The resolution of inflammation is primarily achieved by the depletion of chemokines through matrix metalloproteinases (MMPs)-mediated truncation and sequestration by decoy receptors. This is followed by the induction of neutrophil apoptosis and clearance through macrophage-mediated efferocytosis [33,35]. The proliferative phase is characterized by the formation of the granulation tissue as a result of angiogenesis, extracellular matrix formation and collagen synthesis by proliferating fibroblasts [27,35]. Remodeling is the final phase of wound healing aimed at restoring the structure and function of the skin. Here, fibroblasts are converted to myofibroblasts through transforming growth factor-beta 1 (TGF-β1)-mediated mechanisms [35,36]. Excess ECM and most of the blood vessels are degraded by MMPs and collagen is switched from collagen type III to collagen type I with greater tensile strength [35,37]. This process results in the formation of a fibrous scar that has less flexibility and strength compared to the uninjured tissue. The progression of the remodeling phase is characterized by the reorientation of collagen fibers and recovery of some functional structures of the skin [38]. Completion of dermal remodeling marks the end of the acute wound healing process, which may take varying lengths of time to complete, depending on the degree of injury.

A chronic wound is a persistent wound that fails to heal within three months. The most common chronic wounds are DFUs, pressure ulcers (PUs), and venous leg ulcers (VLUs) [39,40]. While alteration in any of the acute wound healing phases could result in persistent non-healing wounds, a well-regulated inflammatory phase is particularly critical for effective wound healing. Failure to progress from the inflammatory phase to the proliferative phase is a major feature of chronic wounds (Figure 1). Many studies have highlighted the imbalance of the pro-inflammatory and anti-inflammatory cytokines status in chronic wounds [31,32,33,34,35,41,42]. For example, delayed or poor wound healing has been attributed to the perturbation of both the pro-inflammatory responses mediated by the toll-like receptors 2 and 4 (TLR2 and TRL4) as well as the anti-inflammatory activities of interleukin-10 (IL-10) and TGF-β1 [41,42]. Wound infection also hinders the progress of the inflammatory phase to the proliferative phase. Infections accelerate the production of reactive oxygen species (ROS) and advanced glycation end-products (AGEs), escalating the inflammation and oxidative stress at the wound site, thereby delaying wound healing. This issue is of particular concern in DFU patients as they have a higher risk of infections due, in part, to decreased expression of innate anti-microbial peptides, such as β3-defensins (BD3), and impaired neutrophil function and T-lymphocyte responses to infection in a high-glucose microenvironment [43,44,45,46].

## 4. Diabetic Foot Ulcers

**Figure 2:**
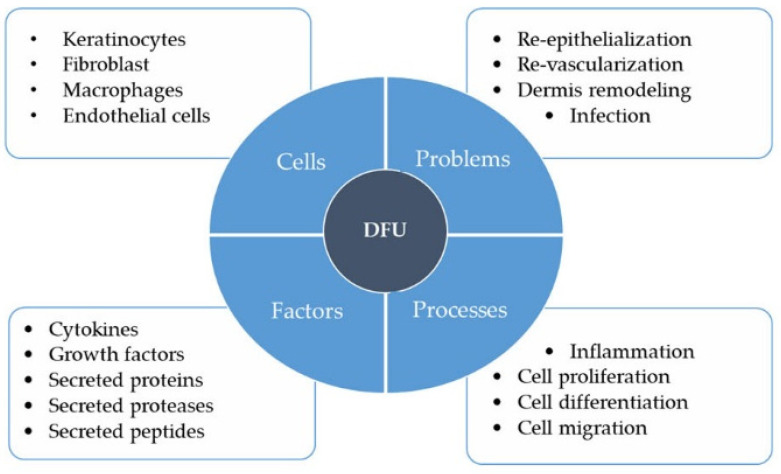
Major factors that contribute to the pathophysiological status of diabetic foot ulcers (DFUs). Keratinocytes, fibroblasts, macrophages, and endothelial cells play important roles in wound healing such as re-epithelialization, revascularization, dermal remodeling, and infection prevention. These functions may be disrupted in the high glucose microenvironment in the skin of diabetic patients, leading to impaired cell proliferation, migration, and differentiation, and prolonged or exaggerated inflammation. Altered cytokine, growth factor, protein, protease, and peptide secretion profiles of different cell types in the skin in response to high glucose microenvironment further define the pathophysiological status of DFUs.

DFUs are one of the most common and severe complications of DM. As a complication arising from a metabolic disorder, the pathophysiological status of DFUs is different from that of other chronic wounds such as PUs and VLUs (Figure 1). A prominent difference between these chronic wounds and DFUs is the high glucose microenvironment. Hyperglycemia is a signature of diabetes and studies have reported that the glucose levels in the skin tissue microenvironment are positively correlated with the plasma glucose level in diabetic patients [47,48,49]. These studies further implicate a higher glucose microenvironment as a major impediment to wound healing at different phases, leading to poorer outcomes for patients with DFU. High microenvironment glucose levels reportedly affect the physiological activities of different cell types in the skin, such as keratinocytes, fibroblasts, macrophages, and endothelial cells that culminate in delayed- or non- healing wounds (Figure 2) [46,50,51,52,53,54,55,56,57,58,59,60,61,62,63,64,65,66,67,68,69,70,71,72].

In the following section, we summarize findings from both in vivo and in vitro studies on how different cell types respond to high glucose concentrations to underscore the unique challenges in treating DFUs and thus the need to develop tailored wound care and treatment strategies for these patients (Figure 2).

### 4.1. Keratinocytes

Basal keratinocytes, the self-renewing stem-like cells of the epidermis, play pivotal roles in multiple processes in wound healing, including the initiation of inflammation and promotion of revascularization, and re-epithelialization. High glucose conditions were shown to perturb keratinocyte functions and negatively impact the resolution of inflammation and re-epithelialization. In diabetic wounds, keratinocytes exhibited increased and sustained expression of inflammatory chemokines such as IL-8, macrophage inhibitory protein-2 (MIP2) and MCP1, leading to persistent neutrophil and macrophage infiltration to the wound bed and non-resolution of the inflammatory phase [50,61,66,67]. Aberrant expression of natural antimicrobial peptides, such as human beta-defensins and cathelicidins by keratinocytes cultured in high glucose and in diabetic animal models, was proposed to contribute to the increased incidence of infection in diabetic wounds [46,68,69]. Exposure of keratinocytes to high glucose conditions inhibited cellular proliferation and triggered aberrant differentiation via the IGFR signalling pathway [73,74]. Re-epithelialization of the wound may also be impacted by reduced keratinocyte migration in high glucose environments due to the inhibition of the p38-MAPK autophagy pathway and decreased expression of MMPs such as MMP-1, to degrade and remodel the collagen-based ECM [70,71,72].

### 4.2. Fibroblasts

During the remodeling phase, fibroblasts proliferate and migrate to the wound site, where they synthesize and secrete ECM components such as fibronectin and collagen to form the structural framework for the granulation tissue. Fibroblasts also secrete growth factors that aid angiogenesis and re-epithelialization as well as strengthening the skin structure. Fibroblasts derived from diabetic ulcers had abnormal cell morphology and showed defects in proliferation and migration in culture [51]. Molecular pathways that promote the motility of fibroblasts, such as miR-21 and JNK signaling, are impaired in diabetic patients and in vitro assays [53,54]. Fibroblasts cultured in high glucose also exhibited transcriptomic changes associated with altered NF-κB, TNF, WNT, and Hippo signaling pathways and production of AGEs and collagen [52,55]. These findings suggest that high glucose conditions in diabetic patients may impair fibroblast functions necessary for the remodeling of the dermal layer during wound healing.

### 4.3. Macrophages

Macrophages derived from circulating monocytes recruited to the wound play essential roles in the inflammatory and proliferative phase of wound healing. A study reported an increase in pro-inflammatory M1 macrophages and decrease in anti-inflammatory M2 macrophages in the serum of diabetic patients [56]. Changes in the ratio of the pro- and anti-inflammatory macrophages corresponded to the increased levels of pro-inflammatory cytokines such as IL-1β, IL-6, and tumor necrosis factor-α (TNF-α) and reduced amounts of the immunomodulator, IL-10 [56]. Culturing macrophages in high glucose media similarly led to amplified levels of pro-inflammatory cytokines following lipopolysaccharide stimulation [57]. Studies also linked high-glucose conditions to the induction of the Nod-like Receptor Family Pyrin Domain Containing 3 (NLRP3) inflammasome and IL-1 in macrophages, derived from THP1-monocytes or diabetic patients [58,59]. The effect of upregulated NLRP3 inflammasome activation at the wound sites contributed to elevated IL-1 and caspase-3 activities and resulted in impaired wound healing in diabetic patients and mouse models [58,60]. Additionally, altered cytokine secretion by macrophages in high glucose conditions may further exacerbate poor healing in DFUs by inhibiting angiogenesis and keratinocyte migration and re-epithelialization [62,63].

### 4.4. Endothelial Cells

During the proliferative phase of healing, angiogenesis facilitates the formation of new blood capillaries, which invade and form a disorganized dense microvascular network in the granulation tissue. Diabetic wounds have been reported to have decreased production of pro-angiogenetic factors, giving rise to lower vascularity and capillary density. This could be partially attributed to the effect of high glucose environment on endothelial cells. For example, down-regulation of miR-126 expression in endothelial cells cultured in high glucose resulted in diminished cellular response to vascular endothelial growth factor (VEGF) signaling, corresponding to the vascular abnormalities observed in miR-126 mutant mice [64,65].

### 4.5. Overall Impact of High Glucose on DFU Wound Healing

Taken together, there is substantial evidence to show that the high-glucose environment in diabetic patients impedes wound healing by altering the functions of keratinocytes, fibroblasts, macrophages, and endothelial cells during wound healing. The DFU wounds are thus largely characterized by abnormal levels of biomolecules such as growth factors, cytokines, proteases and peptides at different phases of healing (Figure 2). These altered cellular and molecular profiles shape the unique pathophysiological status of DFU and distinguish it from other chronic wounds.

## 5. Treatment of Diabetic Ulcer/Foot Ulcer

The current standard care for DFUs focuses on enhancing the wound healing process. DFU management primarily employs the use of active biocompatible wound dressings to aid healing, while skin grafts may be used in the treatment of non-healing or recurrent DFU wounds. In recent years, new therapeutic procedures, including stem-cell-based therapy, have also emerged as prospective treatments for DFU.

### 5.1. Wound Dressings

Biocompatible polymer-based wound dressings are commonly used in DFU wound management. Dressings are usually made from either natural polymers, biocompatible synthetic polymers or a combination of natural and synthetic polymers [75,76]. For example, the Integra^®^ Dermal Regeneration Template consists of a silicone-based outer layer that performs the function of the skin epidermis and an inner layer of bovine tendon collagen and glycosaminoglycan matrix, which provides a scaffold for dermal regeneration [77,78]. The primary functions of these active wound dressings are to keep the wound environment moist and provide a barrier against the infiltration of infectious agents. This helps to facilitate the processes of re-epithelialization and tissue remodeling, while minimizing complications arising from inflammatory responses triggered by microbial infections. The management of chronic DFUs is complicated by higher risks of wound infections and poorer healing rates due to changes in cellular behaviors, growth factor and cytokine secretion in response to a high-glucose microenvironment. Dressings with improved antimicrobial and pro-healing properties are now extensively explored as new treatment options for DFUs [79,80,81].

### 5.2. Skin Grafts

Skin grafting is used to treat severe DFUs that do not improve with the application of wound dressings [82]. There are various types of skin grafting employed in chronic wound treatment. Split-thickness skin grafting (STSG) utilizes autogenic or allogenic skin, consisting of the epidermis and a small portion of dermis, collected from patients or donors. While STSG has demonstrable effectiveness in enhancing wound healing, this treatment method is prone to issues such as non-healing secondary wounds, infection, and graft rejection in diabetic patients that could result in even poorer healing outcomes and increased risks of amputation. Bioengineered human skin equivalent (HSE) products are also used for grafting, and they eliminate the issue of non-healing secondary wounds [83,84,85]. Bioengineered skin substitutes are broadly categorized into epidermal-, dermal- and bilayered-constructs, depending on the cellular composition and thickness of the constructs [86]. Cultured epithelial autografts typically comprise 6-8 stratified layers of autologous keratinocytes grown on irradiated murine fibroblasts. On the other hand, dermal skin substitutes contain allogenic dermal fibroblasts within a collagen-based extracellular matrix scaffold. Bilayered epidermal–dermal skin substitutes contain a tier of stratified keratinocytes in close proximity to a lower layer of dermal fibroblasts and are the most similar in physiology and cellular composition to natural skin. Dermal and bilayered skin substitutes, such as Dermagraft^®^, Apligraft^®^, and OrCel^®^, have been approved for the treatment of DFUs. However, evidence suggests that true engraftment of these bioengineered tissues does not occur and cells in these constructs do not survive for more than 6 weeks [87,88,89]. As the apparent therapeutic benefits of these skin substitutes primarily result from increased pro-healing signaling and cellular crosstalk when the allogenic skin constructs are placed in proximity to the wounds, the mode of action of such skin equivalent grafts may be viewed more appropriately as a short-lived cell-based therapy.

### 5.3. Cell-Based Therapy

In addition to skin-specific cells, the use of stem cells in bioengineered products to enhance the healing of DFUs is an area of intensive research [90,91,92,93,94,95]. Mesenchymal stem cells (MSCs) derived from tissues such as adipose tissues and bone marrow have been used in multiple studies. Of these, bone-marrow-derived MSCs (BM-MSCs) are the best characterized to date with regards to diabetic wound healing in clinical settings [40,96,97,98,99,100,101]. Autologous BM-MSCs administered to wounds accelerated wound closure and revascularization in the dermis and increased the thickness of the wound bed [40,96,97,98,99,100,101]. The therapeutic effects of BM-MSCs include modulation of the inflammatory responses, stimulation of angiogenesis and promotion of proliferation of keratinocytes and fibroblasts by the release of growth factors [102,103,104]. Other MSCs, such as those derived from adipose tissues and peripheral blood, also produced similar therapeutic effects in pre-clinical studies, highlighting their potential in treating chronic wounds in diabetic patients (Table 1).

## 6. Three-Dimensional Bioprinting Approaches to Aid Wound Repair

Three-dimensional (3D) printing is a process in which a user-defined object is fabricated through the deposition of materials in successive layers. Three-dimensional bioprinting is a subset of 3D printing that has been widely used in tissue engineering to recreate biomimetic tissues. This additive manufacturing technique features great flexibility and reproducibility in fabricating mechanically stable biocompatible tissue or organ constructs that mimic the native microenvironment [112]. Broadly, there are three types of 3D bioprinting technologies: inkjet-based, extrusion-based, and light/laser-based. Each of these has specific strengths and weaknesses which make them suitable for different biomanufacturing functions.

### 6.1. Three-Dimensional Bioprinting Techniques

Drop-on-demand (DOD) is the most established inkjet-based bioprinting technology and is sub-categorized into thermal, piezoelectric, and electromagnetic DOD, which each share a similar printing mechanism [113,114]. The printing process consists of two phases: (1) the dispensing of bioink droplets to specific locations on the substrate; and (2) the interaction between the bioink droplets and substrate upon contact (crosslinking and gelation). The printing resolution corresponds to the droplet size and can be optimized by adjusting the nozzle diameter, viscosity of ink, voltage impulse frequency, and, for thermal inkjet, the temperature gradient [115]. The strengths of inkjet bioprinting are low cost, fast printing speed, high resolution, and capabilities of altering concentration gradient [116]. The drawbacks of this technology are low seeding density and compromised cellular viability and functionality resulting from the crosslinking and gelation process [117,118]. Of note, this printing technology has been modified and developed into hand-held portable bioprinting devices for in situ printing applications to tackle the problem of irregular wound topology in conventional skin grafting [119,120].

Extrusion-based bioprinters work by ejecting a continuous stream of biomaterials, instead of droplets, onto the substrate via two types of methods: pneumatic or mechanical [121]. The pneumatic system allows better material flow control by using pressurized gas to extrude the bioink from the nozzle, while the mechanical system provides better spatial control with compressional forces from screws or pistons. While capable of printing constructs at high cell densities, the viability of the extrusion-based printed cells is lower (40–86%) compared to inkjet-based bioprinting (>90%). Lowering the extrusion pressure and increasing nozzle size can improve cell viability but result in reduced printing speed and poorer resolution [118,122]. Hence, fine-tuning of the nozzle diameter, printing pressure, and speed is necessary to ensure the functionality of tissue constructs printed using this method [123]. This printing technology supports an extensive diversity of compatible materials such as hydrogels, biocompatible copolymers and cell spheroids with high seeding density [123], and is commonly used in in vitro bioengineering approaches to generate 3D human skin constructs.

Laser/light-assisted bioprinting (LAB) is a modification of the laser-induced forward transfer (LIFT) technology, which works by focusing a pulsed laser on an energy-absorbing ribbon layer to generate high-pressure bubbles that eject the bioink onto the substrate [116,124]. Thus, compared to inkjet and extrusion-based bioprinting, nozzle clogging is not experienced in LAB, which can, therefore, be used for a variety of bioinks at different viscosities to achieve high-resolution printing [123,125]. However, printing at high-resolution will result in a slow printing rate and impact the hydrogel fidelity and construct functionality due to dehydration [123]. As such, this printing technology is more commonly applied to acellular biomanufacturing such as the incorporation of precise amounts of biomolecules into wound dressings.

Each of the 3D bioprinting techniques has its specific strengths and weaknesses that make it suitable for different biomanufacturing platforms, including in vitro and in situ bioprinting, that can be exploited for the development of better therapeutic options for treatment and care of DFUs.

### 6.2. Application of 3D Bioprinting Approaches to Wound Treatment

#### 6.2.1. In Vitro Bioprinting

A major advantage of bioprinting is the capacity to incorporate different bioinks or cell mixtures in biocompatible matrices into precise spatial orientations or layers in the printed constructs. This feature can be exploited to create skin substitutes with more complex cellular composition and produce constructs that are more similar to the native tissue, to enhance engraftment and the wound healing process.

Most currently available human skin substitutes used in DFU treatment are generated by culturing keratinocytes and fibroblasts on biocompatible scaffolds. In diabetic wounds that have lower levels of pro-angiogenetic signals, these simplistic constructs largely fail to become vascularized or integrate with host tissues, and the transplanted cells thus do not survive for long. To promote vascularization and better tissue integration, 3D bioprinted skin substitutes containing layers of neonatal human dermal fibroblasts and epidermal keratinocytes, and human dermal microvascular endothelial cells (EC)s embedded in fibrin-collagen bioink were generated [126]. These EC-containing skin constructs were transplanted onto full-thickness excision wounds on mice to examine their effects on wound healing. The bioprinted EC-containing skin mimetic graft resulted in accelerated healing of the wound, with 17% improvement in wound contraction, compared to the commercial skin substitute graft and acellular matrix controls. In another study, 3D-printed constructs containing cord blood-derived human ECs, placental pericytes (PC)s, foreskin dermal fibroblasts and keratinocytes in collagen bioink were found to exhibit comparable biological and morphological functions to that of native human skin in vitro [127]. More importantly, when grafted onto wounds in experimental models, rapid invasion of the host microvasculature, the presence of human EC-lined microvessels and a high degree of epidermal organization were observed 2 and 4 weeks post-implantation for the PC and EC-containing grafts. These studies demonstrate that these enhanced 3D-bioprinted skin substitutes can improve graft vascularization and integration, leading to better wound healing outcomes.

Bioprinting is also applied to the development of stem cell-based therapies for DFU, to precisely incorporate active stem cells at desired densities into bioactive scaffolds to create wound patches with enhanced regenerative and wound healing potential. For example, a 3D-printed skin patch containing adipose-derived stem cells (ASCs) and endothelial progenitor cells (EPCs) in decellularized porcine skin ECM was reported to enhance re-epithelialization [128].

These findings demonstrate the applicability of 3D-bioprinted cellular constructs in wound healing by targeting problems such as re-vascularization to improve the long-term survival of the skin substitutes. While additional studies and optimization will be required, the use of 3D-bioprinting to create highly complex cellular constructs with relevant cell types at specific densities and precise spatial distribution will become an important resource to generate novel and targeted DFU treatment.

#### 6.2.2. In Situ Bioprinting

In situ bioprinting capitalizes on the drop-on-demand inkjet technology to print cell-laden bioinks directly onto body sites with the use of hand-held devices or robotic automated systems. This method of bioprinting can be exploited to apply concentrated skin cell suspensions over wounds to accelerate DFU healing through cell–cell signaling, as a viable alternative to the use of skin equivalent substitutes. In a proof-of-concept study, a portable cartridge-based inkjet printer coupled with a laser scanner was used to print suspensions of human fibroblasts and keratinocytes at high densities in fibrin-collagen bioink onto full-thickness excisional wounds in experimental mice [119]. The printed skin constructs on the wounds were found to retain high cellularity, and the presence of human cells can be observed in the tissue for up to 6 weeks post-printing. Wounds treated by direct in situ skin bioprinting were found to heal faster compared to the untreated and acellular controls, with the formation of organized dermis and epidermal sublayers [119].

The use of stem cells was also evaluated for in situ wound healing. Skardal et al. used a robotic inkjet bioprinter to print amniotic fluid-derived stem cells (AFSC)s and BM-MSC-laden fibrin-collagen bioink directly onto full-thickness skin wounds in mice [120]. Compared to the gel-only control, mice treated with AFSCs and BM-MSCs presented greater wound closure and re-epithelialization. In a further experiment to modulate paracrine activities at the wound site, the authors then printed AFSCs-containing, heparin-conjugated hyaluronic acid hydrogels directly onto a full-thickness wound. The hydrogel was found to sequester AFSC-secreted cytokines in situ, prolonging the paracrine activity and led to more rapid closure and vascularization of the full thickness wounds.

A primary advantage of in situ bioprinting is the ability to layer the cell-laden bioinks according to the precise topography of the wound, thus, this method is particularly useful for adaptation to the treatment of wounds with irregular topography. Co-development of cell-based therapies with further optimization of in situ bioprinting techniques will be key to generating better therapeutic options for DFU patients. Table 2 outlines the recent advances in 3D bioprinting approaches in improving the quality and functions of wound management.

### 6.3. Future Directions for 3D Bioprinting Strategies in Diabetic Wound Repair

Moving forward, it is envisioned that 3D bioprinting could play a complementary role in the development of advanced wound scaffolds or skin grafts that enhance the healing process and restore the skin’s structure and functions in the diabetic wound. Here, we highlight some possible avenues of innovation in which 3D bioprinting could be applied to aid DFU management.

#### 6.3.1. Development of Novel Biocompatible ECM-Based Hydrogels as Bioinks

The use of appropriate biocompatible ECM-based hydrogels to create the native-like ECM-based microenvironment in printed skin constructs is important to tailor these skin grafts for DFU treatment. At present, commonly available biocompatible ECM-based hydrogels, both natural and synthetic, do not fully recapitulate the bioactivity of native ECM due to the absence of important biochemical factors. Hence, it is imperative to develop new bioink materials that match the mechanical, rheological, and biological properties of the target tissues.

There are a few approaches to be considered in developing novel bioinks for the DFU-specific bioprinted skin substitutes. One approach being decellularized ECM (dECM)-based bioinks, derived from decellularized tissues of interest that contain the required growth factors and cytokines to cue cellular growth and differentiation [130,131]. Apart from dECM, materials that respond to external stimuli can also be used as bioinks. These materials can undergo conformational changes in response to different physiological triggers in the microenvironment, making them favorable for tissue regeneration and drug delivery. An example of stimulus is the cell traction force, which has been exploited to promote vascular network formation in bioprinted tissues and wound repair [132,133]. As chronic wounds are predominantly more alkaline (5.4–7.4) than healthy skin (4.7–5.75) due to defective ECM and the presence of microbes, pH-responsive bioinks may also be useful for enhancing wound repair [134]. For instance, a pH-responsive hydrogel has been developed to release sequestered vascular endothelial growth factor (VEGF) at pH 7.4 instead of pH 5 and 6 [135]. Such bio-responsive bioinks can be adapted to recreate the most optimal microenvironment to promote healing in DFUs.

#### 6.3.2. Personalized Treatment

Three-dimensional printing facilitates the development of precision medicine by tailoring to patient-specific anatomical features and disease profiles. Examples of personalized tissues that have been manufactured by 3D bioprinting include cartilage [17], bone [18], as well as cardiac patches [19]. In comparison to commercially available skin substitutes, 3D bioprinting of skin grafts enables customization of the size and shape of constructs to fit the patient’s unique wound topology, thereby ensuring complete wound coverage and better aesthetics post-healing [136]. This feature is elegantly illustrated through in situ bioprinting, where the material is directly deposited onto the wound site to achieve complete coverage [119,120]. However, at present, in situ bioprinting is only achievable for small wounds (<3 × 3 cm). For wounds with larger surface areas, cell proliferation, tissue maturation and vasculature formation within the bioprinted skin construct will be necessary to maintain the graft viability in vivo [137]. This can potentially be achieved by first printing the skin construct in vitro, where a construct of a specific size, geometry and cellular composition is fabricated based on a 3D scan of the graft area on the patient before transplanting onto the wound. In addition, personalized drug-laden wound dressings or skin grafts could be manufactured by 3D bioprinting to enable the administration of specific dosages or combinations of therapeutic compounds, such as anti-inflammatory drugs, to the wound sites to address patient-specific therapeutic regimens [138].

#### 6.3.3. Infusion of Bioactives in Acellular 3D-Printed Wound Dressings

In addition to true 3D-bioprinting applications in which tissue-like mimetics are printed using cell-laden bioinks, 3D printing techniques can also be applied in the fabrication of better therapeutic products for DFU management. For example, the flexibility and precision of 3D printing techniques have been capitalized upon to incorporate bioactive compounds into wound dressings tailored for DFU-specific treatments to enhance their anti-microbial and pro-healing properties. Recent developments include wound dressings that were 3D-printed using biodegradable polydimethylsiloxane infused with silicon oil and silver nanoparticles. These dressings showed highly effective antibacterial activity and improved re-epithelialization and granulation tissue formation when applied onto a full-thickness wound inoculated with *S. aureus* and *E. coli* in animal models [129].

Re-epithelialization, dermal remodeling, and angiogenesis during wound healing are stimulated by bioactive molecules such as secreted proteins, growth factors, and cytokines synthesized and secreted by physiologically healthy cells. In DFUs, the synthesis and secretion of these biomolecules are altered due to impaired cell functions in response to the high-glucose microenvironment. To improve the commercially available bioartificial dermal template or scaffolds for DFU wound management, multiple growth factors can be incorporated into the products [139,140,141,142]. For example, the inclusion and delivery of VEGF in 3D-bioprinted scaffolds has been shown to increase the proliferation of endothelial cells and vascularization in vivo [141,142]. These results demonstrate the potential of using bioprinted wound scaffolds containing VEGF or other growth factors to restore blood supply to the wound site to facilitate healing. The precise spatial incorporation of bioactive molecules into novel biocompatible ECM by 3D bioprinting technologies can be further exploited to incorporate temporal release and dosage control of the bioactive molecules to further tailor these dressings for DFU management.

## 7. Conclusions

The demand for DFU wound management is expected to increase following the rising incidence of DM patients. It is imperative to develop DFU-specific therapeutic tools to improve current DFU wound management so that the increasing demands do not over-strain the limited healthcare resources. Compelling evidence has demonstrated that the integration of various 3D bioprinting approaches with existing wound treatments can facilitate the development of novel and improved wound healing strategies. Further co-development of 3D-bioprinting technologies and promising novel approaches, such as personalized drug-coated dressings and stem cell-based therapeutics, will be key to address the specific pathophysiological challenges that DFUs present.

## Figures and Tables

**Table 1:** Current and experimental treatments for DFUs.

Treatments/Key Examples	Mechanisms	Effects
Wound dressings Natural Polymer (Promogran, collagen) Synthetic polymer (Polyglactin, 45S5 Bioglass (PLGA)) Combination of natural and synthetic polymer (Integra^®^ Dermal Regeneration Template)	Increase MMP-2, and MMP-9 in the epidermis [105]. Promote secretion angiogenic growth factors [106,107].	Rapid wound healing Promote re-epithelialization Revascularization and angiogenesis
Skin grafting Split-thickness skin graft (STSG) Human skin equivalent (HSE) (Dermagraft^®^, Apligraft^®^, OrCel^®^)	Increase MMP-2, and MMP-9 in epidermis and dermal TIMPs activity [108]. Increase cytokines and growth factors [84,85,87]. Deliver growth factors and extracellular matrix proteins to the wound bed [109].	Improve wound healing
Stem-cell therapy Bone-marrow derived MSCs Umbilical cord blood-derived MSCs Adipose-derived MSCs Placenta-derived MSCs Amniotic fluid-derived MSCs	Promote secretion of angiogenic factors [110]. Increase the expression of keratinocyte-specific proteins and cytokeratin [110]. Increased collagen synthesis [91,94]. Modulation of immunological abnormality [111].	Promote wound healing Increase re-epithelialization and cellularity Promote dermal collagen synthesis Vascular regeneration

**Table 2:** Summary of 3D bioprinting approaches to aid in wound healing.

Bioprinting Devices	Biomaterials for Printing and Construct Applications	Outcomes
In vitro 1. Robotic, inkjet motor-driven device	Bioink: Fibrin and collagen Cells: Neonatal human dermal fibroblast, human dermal microvascular endothelial cells and neonatal human epidermal keratinocytes Application: Full-thickness skin excision (1.7 cm × 1.7 cm) on athymic nude mice	Bioprinted skin graft showed accelerated wound healing with 17% improvement in wound contraction and formation of microvasculature [126].
2. Robotic, inkjet and extrusion device (in-house built)	Bioink: Decellularized extracellular matrix of porcine skin tissue Cells: Adipose-derived stem cells (ASCs) + endothelial progenitor cells (EPCs) Application: 10 mm biopsy punch full-thickness excisional wound on BALB/c A-nu/nu	Bioprinted skin patch remarkably enhanced neovascularization as well as wound closure and re-epithelization. Cell-printed dECM patch also exhibited better-wound healing activity compared to only ASC/EPCs mixture [128].
3. Robotic, extrusion pressure-driven device	Bioink: Rat tail type I collagen Cells (dermis): human foreskin dermal fibroblasts, human endothelial cells derived from cord blood endothelial colony-forming cells and placental pericytes (PCs) Cells (epidermis): Human foreskin keratinocytes Application: Excised dorsal mouse skin	Skin substitutes formulated with human ECs and PCs contained vascular structures 2-weeks post-engraftment and a higher degree of epidermal organization. Grafts containing PCs in addition to ECs appeared to evoke a more extensive angiogenic host response [127].
In situ 1. Robotic, inkjet pressure-driven device (XYZ movement system with interchangeable printhead and laser scanner)	Bioink: Fibrin and collagen Cells: Human fibroblasts and keratinocytes Application: Full-thickness excisional skin defect (3 × 2.5 cm) on female outbred nu/nu mice	Bioprinted constructs promoted accelerated wound closure with fully formed and organized dermis and epidermis. Fibroblasts and keratinocytes remained 6 weeks post engraftment [119].
2. Robotic, inkjet pressure-driven device (Three-axes movement system with pressure-driven nozzles and an optical sensor)	Bioink: Fibrin and collagen Cells: Amniotic fluid-derived stem cells (AFS) and Bone marrow-derived mesenchymal stem cells (MSC) Application: Full-thickness skin wound (2.0 × 2.0 cm) on nu/nu mice.	Wounds treated with AFS and MSC cells had faster-wound closure, re-epithelialization and better neovascularization than cell-free controls [129].
3. Robotic, inkjet pressure-driven device (Three-axes movement system with pressure-driven nozzles and swappable hydrogel cartridges in-line with back-pressure and print nozzle)	Bioink: Heparin-conjugated hyaluronic acid (HA) and thiolated HA Cells: Amniotic fluid-derived stem (AFS) cells Application: Full-thickness skin wound (2.0 × 2.0 cm) on nu/nu mice.	HA-HP hydrogel supports the extended release of AFS-secreted cytokines by heparin-associated sequestration that improved wound closure, re-epithelialization, and vascularization [120].
